# Clinical reports of pulmonary metastasectomy for colorectal cancer: a citation network analysis

**DOI:** 10.1038/sj.bjc.6606060

**Published:** 2011-03-08

**Authors:** F Fiorentino, C Vasilakis, T Treasure

**Affiliations:** 1 Clinical Operational Research Unit, University College London, London WC1H 0BT, UK

**Keywords:** colorectal cancer, pulmonary mestastasectomy, citation network analysis, hepatic metastasectomy, history of medicine

## Abstract

**Introduction:**

Pulmonary metastasectomy for colorectal cancer is a commonly performed and well-established practice of ∼50 years standing. However, there have been no controlled studies, randomised or otherwise. We sought to investigate the evidence base that has been used in establishing its status as a standard of care.

**Methods:**

Among 51 papers used in a recent systematic review and quantitative synthesis, a citation network analysis was performed. A total of 344 publications (the 51 index papers and a further 293 cited in them) constitute the citation network.

**Results:**

The pattern of citation is that of a citation cascade. Specific analyses show the frequent use of historical or landmark papers, which add authority. Papers expressing an opposing viewpoint are rarely cited.

**Conclusions:**

The citation network for this common and well-established practice provides an example of selective citation. This pattern of citation tends to escalate belief in a clinical practice even when it lacks a high-quality evidence base and may create an impression of more authority than is warranted.

There is a professional consensus regarding the effectiveness of pulmonary metastasectomy for colorectal cancer (PMCRC), and along with metastasectomy for other primary sites, this surgery has become a routine (Internullo *et al*, 2007). In the course of a systematic review of pulmonary metastasectomy for this disease, 51 publications (listed in Appendix A) were identified, which contained data on case selection and outcomes (Fiorentino *et al*, 2010). These 51 surgical follow-up studies were published from 1971 to 2006; they include a total of 3504 patients who were operated on over a 40-year period starting in the mid 1960s. Five-year survival is reported at a rate of approximately 30–50% and is attributed to the beneficial effects of metastasectomy. The more recent papers in the review cite earlier papers, and in addition, 293 other clinical reports are cited (listed in Appendix B).

Citation network analysis is recommended as a standard tool in evidence-based medicine (EBM) (Haynes *et al*, 2006). The reason for this is well illustrated by Greenberg (2009) who in the *British Medical Journal* showed how authors seeking a basis for their own practice may create a citation cascade ‘resulting in unfounded authority of claims’. *New Scientist*, concerned with bias in the citation of stem cell research, used citation network analysis to show that US scientists rarely cited non-US authors (Aldhous, 2010). In the course of sifting, reading, and extracting data for analysis, we became aware of the absence of controlled trials regarding metastasectomy, as have others (Pfannschmidt *et al*, 2007, 2010). These published clinical reports lack data on how such patients would have fared had they not undergone pulmonary metastasectomy. In the course of planning a randomised controlled trial to address the matter (Treasure *et al*, 2009, 2010) and in line with EBM recommendations, we undertook a citation network analysis of papers included in our review.

## Methods

We examined the reference lists of the 51 papers incorporated into a systematic review and quantitative synthesis (SRQS) (Fiorentino *et al*, 2010). We first excluded standard references to statistical methods and to books and book chapters. All the remaining citations were individually characterised by two of the authors (TT and FF). We defined 4 categories of particular interest: clinical follow-up studies of PMCRC, reports regarding hepatic metastasectomy, historical references, papers questioning the practice, and 10 other categories, including epidemiology, adjuvant therapies, pathological studies, and surgical techniques.

We then constructed a citation network representing all papers as nodes and citations as links from one node to another (Greenberg, 2009). A custom-designed relational database (MS Access, Microsoft, Redmond, WA, USA) was used to store and query the citation network data. We used Pajek, a freely available special-purpose social network analysis software tool (available from http://pajek.imfm.si/doku.php?id=pajek/), to perform visualisations of the citation networks (de Nooy *et al*, 2005).

## Results

The 51 follow-up reports included in the SRQS were published as follows: 1 in 1971, 4 in the 1980s, 19 in the 1990s, and 27 between 2000 and 2006. These papers each cited between 10 and 36 references to a total of 1132. After planned exclusions, there were 972 citations to 293 other unique publications. The frequency distribution of citations was markedly skewed with 203 of 344 cited only once. The 5 most referenced papers were cited 31, 30, 27, and 2 × 22 times (McCormack and Martini, 1979; Mansel *et al*, 1986; Goya *et al*, 1989; McAfee *et al*, 1992; Okumura *et al*, 1996).

**Figure 1:**
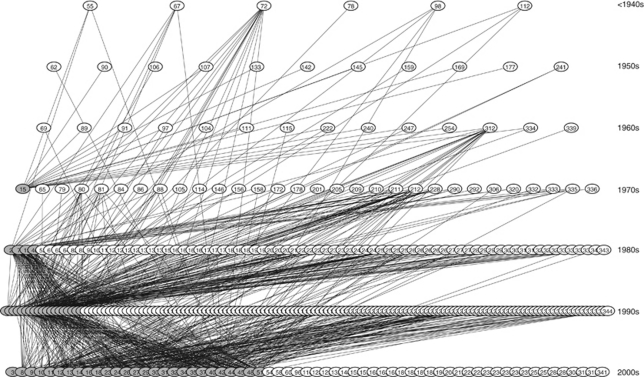
All papers included in the citation network by decade of publication. The 51 index papers are in grey and are listed in Appendix A. The 293 cited papers are listed in Appendix B.

The network of the citations of 51 SRQS papers to 344 references in the network is illustrated in Figure 1 following the method of Greenberg. A cascade such as the one he described can be discerned.

**Figure 2:**
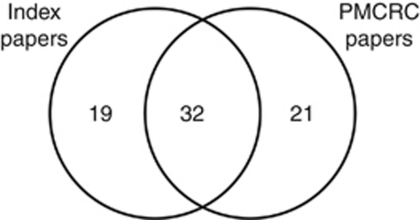
Venn diagram of 51 ‘index papers’ from the systematic review and quantitative synthesis (Fiorentino *et al*, 2010) and 53 papers categorised in the citation network as reports of clinical series of pulmonary metastasectomy for colorectal cancer (PMCRC) and their overlap. The total is 72 publications.

**Figure 3:**
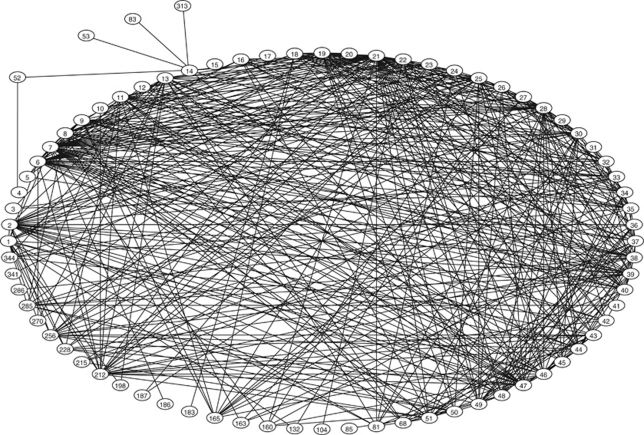
This network includes all the 72 papers in Figure 2 showing their numerous citations to each other. On the periphery are the four questioning papers. The numbers refer to papers listed papers in Appendix A and Appendix B.

Of the 51 papers in the systematic review, 32 were classified for citation analysis as exclusively concerning and supporting PMCRC (both citing and cited), 19 were otherwise classified (e.g., mixed series of hepatic and pulmonary metastasectomy), and 21 further PMCRC papers were cited by SRQS papers (Figure 2). The citations among these 72 papers are shown in Figure 3 and in addition, the 4 papers questioning practice. Of these, for example, Aberg *et al* (1980) and Aberg (1997) argued that apparent longer-than-expected survival is a matter of case selection. Two other authors expressed caution attributing survival to surgery (Casciato *et al*, 1983; Todd, 1997). These are rarely cited compared with the dense network of citations between the index papers and other supportive PMCRC publications.

## Discussion

Our search of the literature for evidence regarding PMCRC was prompted in part by a NICE Cancer Services Manual update stating, ‘Surgery for metastases confined to the liver or lung can be curative…for a minority of patients, it can increase five-year survival rates from close to zero to over 30%’ (National Institute for Health and Clinical Excellence (NICE), 2004). We speculated as to the evidence for this statement, but the single reference cited contained no information regarding pulmonary metastasectomy (Stangl *et al*, 1994). This is a clear instance of not only selective but also inappropriate citation.

The manner by which ‘history’ is cited is of interest (Pastorino and Treasure, 2010). In our citation analysis, we categorised four papers as historical. As an example, the report by Blalock (1944) in the 1944 *New England Journal of Medicine* is cited by 14 of 51 of the index papers and is the thirteenth most frequent of 334 cited papers. Typical citations to it are: ‘The role of pulmonary metastasectomy for colorectal carcinoma was first introduced in 1944.’ (Patel *et al*, 2003) and ‘Since Blalock reported the first pulmonary resection for colorectal metastases in 1944, lung metastases…have been considered to be cured by resection in selected cases.’(Ike *et al*, 2002). In summarising a guest lecture to The Massachusetts Medical Society, Blalock wrote ‘It is only eleven years since the first one stage removal of an entire lung...’. He was informing his colleagues that pneumonectomy is achievable; the fact that it was for a metastasis was coincidental. He tells us nothing about control of colorectal cancer or the eventual outcome beyond recovery from the operation. Furthermore, in current practice, pneumonectomy is considered an inappropriate operation for metastasectomy (Migliore *et al*, 2010).

Blalock's account of a pneumonectomy has no relevance to the current practice of pulmonary metastasectomy, and is not even claimed to be a first; hence, why is this such a popular citation? A possible answer is that among surgeons, his name adds authority: he went on to become extremely famous for the Blalock-Taussig shunt to palliate cyanotic heart disease (Blalock and Taussig, 1945). His paper, which would not have been found by a formal search for metastasectomy but is passed on as folklore, gains a significance that Blalock did not intend.

In marked contrast, Aberg's publications are barely mentioned. His paper in 1980 and editorial in 1997 which challenge the effect of pulmonary metastasectomy are cited by only two of the index papers, and yet, unlike Blalock's paper, Aberg's publications have metastasectomy in their title and could not be missed in a literature search. The failure to cite suggests bias against his paper rather than that it was not retrievable. In addition, US citations may have a higher perceived status (Link, 1998). Blalock was from a prestigious American institution, Johns Hopkins, whereas Aberg worked in a less well-known Swedish hospital.

To quote from a remarkable essay on the whole question of citation (MacRoberts and MacRoberts, 1996), ‘The cumulative effect of citing more and more people who similarly agree with the author is to concretize the universality of the knowledge claim.’ Greenberg describes information cascades: authors write their clinical experience, citing similar practices and thus gain affirmation. However, it is like rolling a snowball: it gets bigger and bigger – but it is just more snow.

## Appendix A

The 51 publications that were entered in the quantitative synthesis are as follows:

Fiorentino F, Hunt I, Teoh K, Treasure T, Utley M (2010) Pulmonary metastasectomy in colorectal cancer: a systematic review and quantitative synthesis. *J R Soc Med* **103:** 60–66.

The citation numbers 1–51 relate to those in Figures 1 and 3

(1) Baron O, Hamy A, Roussel JC, Galetta D, Al HO, Duveau D *et al* (1999) Surgical treatment of pulmonary metastases of colorectal cancers. 8-year survival and main prognostic factors. *Rev Mal Respir* **16**(5): 809–815.

(2) Brister SJ, deVarennes B, Gordon PH, Sheiner NM, Pym J (1988) Contemporary operative management of pulmonary metastases of colorectal origin. *Dis Colon Rectum* **31**(10): 786–792.

(3) Casali C, Stefani A, Storelli E, Morandi U (2006) Prognostic factors and survival after resection of lung metastases from epithelial tumours. *Interact Cardiovasc Thorac Surg* **5**(3): 317–321.

(4) Cheng LC, Chiu CS, Lee JW (1998) Surgical resection of pulmonary metastases. *J Cardiovasc Surg (Torino)* **39**(4): 503–507.

(5) Fujisawa T, Yamaguchi Y, Saitoh Y, Sekine Y, Iizasa T, Mitsunaga S *et al* (1996) Factors influencing survival following pulmonary resection for metastatic colorectal carcinoma. *Tohoku J Exp Med* **180**(2): 153–160.

(6) Girard P, Ducreux M, Baldeyrou P, Rougier P, Le Chevalier T, Bougaran J *et al* (1996) Surgery for lung metastases from colorectal cancer: analysis of prognostic factors. *J Clin Oncol* **14**(7): 2047–2053.

(7) Goya T, Miyazawa N, Kondo H, Tsuchiya R, Naruke T, Suemasu K (1989) Surgical resection of pulmonary metastases from colorectal cancer. 10-year follow-up. *Cancer* **64**(7): 1418–1421.

(8) Higashiyama M, Kodama K, Higaki N, Takami K, Murata K, Kameyama M *et al* (2003) Surgery for pulmonary metastases from colorectal cancer: the importance of prethoracotomy serum carcinoembryonic antigen as an indicator of prognosis. *Jpn J Thorac Cardiovasc Surg* **51**(7): 289–296.

(9) Iizasa T, Suzuki M, Yoshida S, Motohashi S, Yasufuku K, Iyoda A *et al* (2006) Prediction of prognosis and surgical indications for pulmonary metastasectomy from colorectal cancer. *Ann Thorac Surg* **82**(1): 254–260.

(10) Ike H, Shimada H, Togo S, Yamaguchi S, Ichikawa Y, Tanaka K (2002) Sequential resection of lung metastasis following partial hepatectomy for colorectal cancer. *Br J Surg* **89**(9): 1164–1168.

(11) Inoue M, Ohta M, Iuchi K, Matsumura A, Ideguchi K, Yasumitsu T *et al* (2004) Benefits of surgery for patients with pulmonary metastases from colorectal carcinoma. *Ann Thorac Surg* **78**(1): 238–244.

(12) Irshad K, Ahmad F, Morin JE, Mulder DS (2001) Pulmonary metastases from colorectal cancer: 25 years of experience. *Can J Surg* **44**(3): 217–221.

(13) Ishikawa K, Hashiguchi Y, Mochizuki H, Ozeki Y, Ueno H (2003) Extranodal cancer deposit at the primary tumor site and the number of pulmonary lesions are useful prognostic factors after surgery for colorectal lung metastases. *Dis Colon Rectum* **46**(5): 629–636.

(14) Jaklitsch MT, Mery CM, Lukanich JM, Richards WG, Bueno R, Swanson SJ *et al* (2001) Sequential thoracic metastasectomy prolongs survival by re-establishing local control within the chest. *J Thorac Cardiovasc Surg* **121**(4): 657–667.

(15) Joseph WL, Morton DL, Adkins PC (1971) Prognostic significance of tumor doubling time in evaluating operability in pulmonary metastatic disease. *J Thorac Cardiovasc Surg* **61**(1): 23–32.

(16) Kanemitsu Y, Kato T, Hirai T, Yasui K (2004) Preoperative probability model for predicting overall survival after resection of pulmonary metastases from colorectal cancer. *Br J Surg* **91**(1): 112–120.

(17) Kodama K, Doi O, Higashiyama M, Tatsuta M, Iwanaga T (1991) Surgical management of lung metastases. Usefulness of resection with the neodymium:yttrium-aluminum-garnet laser with median sternotomy. *J Thorac Cardiovasc Surg* **101**(5): 901–908.

(18) Koga R, Yamamoto J, Saiura A, Yamaguchi T, Hata E, Sakamoto M (2006) Surgical resection of pulmonary metastases from colorectal cancer: four favourable prognostic factors. *Jpn J Clin Oncol* **36**(10): 643–648.

(19) Mansel JK, Zinsmeister AR, Pairolero PC, Jett JR (1986) Pulmonary resection of metastatic colorectal adenocarcinoma. A ten-year experience. *Chest* **89**(1): 109–112.

(20) Marincola FM, Mark JB (1990) Selection factors resulting in improved survival after surgical resection of tumors metastatic to the lungs. *Arch Surg* **125**(10): 1387–1392.

(21) McAfee MK, Allen MS, Trastek VF, Ilstrup DM, Deschamps C, Pairolero PC (1992) Colorectal lung metastases: results of surgical excision. *Ann Thorac Surg* **53**(5): 780–785.

(22) McCormack PM, Burt ME, Bains MS, Martini N, Rusch VW, Ginsberg RJ (1992) Lung resection for colorectal metastases. 10-year results. *Arch Surg* **127**(12):1403–1406.

(23) Mineo TC, Ambrogi V, Tonini G, Bollero P, Roselli M, Mineo D *et al* (2003) Long-term results after resection of simultaneous and sequential lung and liver metastases from colorectal carcinoma. *J Am Coll Surg* **197**(3): 386–391.

(24) Moore KH, McCaughan BC (2001) Surgical resection for pulmonary metastases from colorectal cancer. *ANZ J Surg* **71**(3): 143–146.

(25) Mori M, Tomoda H, Ishida T, Kido A, Shimono R, Matsushima T *et al* (1991) Surgical resection of pulmonary metastases from colorectal adenocarcinoma. Special reference to repeated pulmonary resections. *Arch Surg* **126**(10): 1297–1301.

(26) Negri F, Musolino A, Cunningham D, Pastorino U, Ladas G, Norman AR (2004) Retrospective study of resection of pulmonary metastases in patients with advanced colorectal cancer: the development of a preoperative chemotherapy strategy. *Clin Colorectal Cancer* **4**(2): 101–106.

(27) Ogata Y, Matono K, Hayashi A, Takamor S, Miwa K, Sasatomi T *et al* (2005) Repeat pulmonary resection for isolated recurrent lung metastases yields results comparable to those after first pulmonry resection in colorectal cancer. *World J Surg* **29:** 363–368.

(28) Okumura S, Kondo H, Tsuboi M, Nakayama H, Asamura H, Tsuchiya R *et al* (1996) Pulmonary resection for metastatic colorectal cancer: experiences with 159 patients. *J Thorac Cardiovasc Surg* **112**(4): 867–874.

(29) Patel NA, Keenan RJ, Medich DS, Woo Y, Celebrezze J, Santucci T *et al* (2003) The presence of colorectal hepatic metastases does not preclude pulmonary metastasectomy. *Am Surg* **69**(12): 1047–1053.

(30) Pfannschmidt J, Muley T, Hoffmann H, Dienemann H (2003) Prognostic factors and survival after complete resection of pulmonary metastases from colorectal carcinoma: experiences in 167 patients. *J Thorac Cardiovasc Surg* **126**(3): 732–739.

(31) Reddy RH, Kumar B, Shah R, Mirsadraee S, Papagiannopoulos K, Lodge P *et al* (2004) Staged pulmonary and hepatic metastasectomy in colorectal cancer—is it worth it? *Eur J Cardiothorac Surg* **25**(2): 151–154.

(32) Rena O, Casadio C, Viano F, Cristofori R, Ruffini E, Filosso PL *et al* (2002) Pulmonary resection for metastases from colorectal cancer: factors influencing prognosis. Twenty-year experience. *Eur J Cardiothorac Surg* **21**(5): 906–912.

(33) Saclarides TJ, Krueger BL, Szeluga DJ, Warren WH, Faber LP, Economou SG (1993) Thoracotomy for colon and rectal cancer metastases. *Dis Colon Rectum* **36**(5): 425–429.

(34) Saito Y, Omiya H, Kohno K, Kobayashi T, Itoi K, Teramachi M *et al* (2002) Pulmonary metastasectomy for 165 patients with colorectal carcinoma: a prognostic assessment. *J Thorac Cardiovasc Surg* **124**(5): 1007–1013.

(35) Sakamoto T, Tsubota N, Iwanaga K, Yuki T, Matsuoka H, Yoshimura M (2001) Pulmonary resection for metastases from colorectal cancer. *Chest* **119**(4): 1069–1072.

(36) Scheele J, Altendorf-Hofmann A, Stangl R, Gall FP (1990) Pulmonary resection for metastatic colon and upper rectum cancer. Is it useful? *Dis Colon Rectum* **33**(9): 745–752.

(37) Shiono S, Ishii G, Nagai K, Yoshida J, Nishimura M, Murata Y *et al* (2005) Histopathologic prognostic factors in resected colorectal lung metastases. *Ann Thorac Surg* **79**(1): 278–282.

(38) Shirouzu K, Isomoto H, Hayashi A, Nagamatsu Y, Kakegawa T (1995) Surgical treatment for patients with pulmonary metastases after resection of primary colorectal carcinoma. *Cancer* **76**(3): 393–398.

(39) van Halteren HK, van Geel AN, Hart AA, Zoetmulder FA (1995) Pulmonary resection for metastases of colorectal origin. *Chest* **107**(6): 1526–1531.

(40) Vogelsang H, Haas S, Hierholzer C, Berger U, Siewert JR, Prauer H (2004) Factors influencing survival after resection of pulmonary metastases from colorectal cancer. *Br J Surg* **91**(8): 1066–1071.

(41) Wade TP, Virgo KS, Li MJ, Callander PW, Longo WE, Johnson FE (1996) Outcomes after detection of metastatic carcinoma of the colon and rectum in a national hospital system. *J Am Coll Surg* **182**(4): 353–361.

(42) Wang CY, Hsie CC, Hsu HS, Wu YC, Hsu WH, Huang BS *et al* (2002) Pulmonary resection for metastases from colorectal adenocarcinomas. *Zhonghua Yi Xue Za Zhi (Taipei)* **65**(1): 15–22.

(43) Watanabe M, Deguchi H, Sato M, Ozeki Y, Tanaka S, Izumi Y *et al* (1998) Midterm results of thoracoscopic surgery for pulmonary metastases especially from colorectal cancers. *J Laparoendosc Adv Surg Tech A* **8**(4): 195–200.

(44) Watanabe I, Arai T, Ono M, Sugito M, Kawashima K, Ito M *et al* (2003) Prognostic factors in resection of pulmonary metastasis from colorectal cancer. *Br J Surg* **90**(11): 1436–1440.

(45) Welter S, Jacobs J, Krbek T, Poettgen C, Stamatis G (2007) Prognostic impact of lymph node involvement in pulmonary metastases from colorectal cancer. *Eur J Cardiothorac Surg* **31**(2): 167–172.

(46) Wilking N, Petrelli NJ, Herrera L, Regal AM, Mittelman A (1985) Surgical resection of pulmonary metastases from colorectal adenocarcinoma. *Dis Colon Rectum* **28**(8): 562–564.

(47) Yano T, Hara N, Ichinose Y, Yokoyama H, Miura T, Ohta M (1993) Results of pulmonary resection of metastatic colorectal cancer and its application. *J Thorac Cardiovasc Surg* **106**(5): 875–879.

(48) Yedibela S, Klein P, Feuchter K, Hoffmann M, Meyer T, Papadopoulos T *et al* (2006) Surgical management of pulmonary metastases from colorectal cancer in 153 patients. *Ann Surg Oncol* **13**(11): 1538–1544.

(49) Zanella A, Marchet A, Mainente P, Nitti D, Lise M (1997) Resection of pulmonary metastases from colorectal carcinoma. *Eur J Surg Oncol* **23**(5): 424–427.

(50) Zapatero J, Flandes J, Lago J, Devesa M, Glope A, Candelas J (1994) Prognostic factors in pulmonary metastases from colorectal cancer. *Respiration* **61**(5): 280–282.

(51) Zink S, Kayser G, Gabius HJ, Kayser K (2001) Survival, disease-free interval, and associated tumor features in patients with colon/rectal carcinomas and their resected intra-pulmonary metastases. *Eur J Cardiothorac Surg* **19**(6): 908–913.

## Appendix B

Citations 52–344, which all appear in the citation network diagrams of Figure 1 and some in Figure 3, are listed herein. These are 293 publications on clinical material cited by the authors of the 51 papers in the systematic review and quantitative synthesis (Fiorentino *et al*, 2010) in addition to citations made to each other's work.

(52) Aberg T, Malmberg KA, Nilsson B, Nou E (1980) The effect of metastasectomy: fact or fiction? *Ann Thorac Surg* **30**(4): 378–384.

(53) Aberg T (1997) Selection mechanisms as major determinants of survival after pulmonary metastasectomy. *Ann Thorac Surg* **63**(3): 611–612.

(54) Adam R, Avisar E, Ariche A, Giachetti S, Azoulay D, Castaing D *et al* (2001) Five-year survival following hepatic resection after neoadjuvant therapy for nonresectable colorectal. *Ann Surg Oncol* **8**(4): 347–353.

(55) Alexander J, Haight C (1947) Pulmonary resection for solitary metastatic sarcomas and carcinomas. *Surg Gynecol Obstet* **85:**129–146.

(56) Amato A, Pescatori M, Butti A (1991) Local recurrence following abdominoperineal excision and anterior resection for rectal carcinoma 5. *Dis Colon Rectum* **34**(4): 317–322.

(57) Ambiru S, Miyazaki M, Ito H, Nakagawa K, Shimizu H, Nakajima N (1999) Adjuvant regional chemotherapy after hepatic resection for colorectal metastases. *Br J Surg* **86**(8): 1025–1031.

(58) Ambrogi V, Paci M, Pompeo E, Mineo TC (2000) Transxiphoid video-assisted pulmonary metastasectomy: relevance of helical computed tomography occult lesions. *Ann Thorac Surg* **70**(6): 1847–1852.

(59) American Cancer Society (1994) *Cancer Facts and Figures* – *1994*. Atlanta, GA. Ref Type: Report

(60) American Cancer Society (2004) *Cancer Facts and Figures* – *2004*. Atlanta, ACS. Ref Type: Report

(61) American Joint Committee for Cancer Staging and Edn Results Reporting (1987) *Manual for Staging of Cancer 1987*. Chicago, NCI. Ref Type: Report

(62) Astler V, Coller F (1954) The prognostic significance of direct extension of carcinoma of the colon and rectum. *Ann Surg* **139**(6): 846–852.

(63) August DA, Ottow RT, Sugarbaker PH (1984) Clinical perspective of human colorectal cancer metastasis. *Cancer Metastasis Rev* **3**(4): 303–324.

(64) Baldeyrou P, Lemoine G, Zucker JM, Schweisguth O (1984) Pulmonary metastases in children: the place of surgery. A study of 134 patients. *J Pediatr Surg* **19**(2): 121–125.

(65) Ballantine TV, Wiseman NE, Filler RM (1975) Assessment of pulmonary wedge resection for the treatment of lung metastases. *J Pediatr Surg* **10**(5): 671–676.

(66) Ballantyne GH, Quin J (1993) Surgical treatment of liver metastases in patients with colorectal cancer. *Cancer* **71**(12 Suppl): 4252–4266.

(67) Barney J, Churchill E (1939) Adenocarcinoma of the kidney with metastasis to the lung cured by nephrectomy and lobectomy. *J Urol* **42:** 269–276.

(68) Baron O, Amini M, Duveau D, Despins P, Sagan CA, Michaud JL (1996) Surgical resection of pulmonary metastases from colorectal carcinoma. Five-year survival and main prognostic factors. *Eur J Cardiothorac Surg* **10**(5): 347–351.

(69) Bengmark S, Hafstrom L (1969) The natural history of primary and secondary malignant tumors of the liver. I. The prognosis for patients with hepatic metastases from colonic and rectal carcinoma by laparotomy. *Cancer* **23**(1): 198–202.

(70) Bines S, Kind G, Doolas A (1990) Resecting liver metastases. *Probl Gen Surg* **7:** 428–432.

(71) Bismuth H, Adam R, Levi F, Farabos C, Waechter F, Castaing D *et al* (1996) Resection of nonresectable liver metastases from colorectal cancer after neoadjuvant chemotherapy. *Ann Surg* **224**(4): 509–520.

(72) Blalock A (1944) Recent advances in surgery. *N Engl J Med* **231:** 261–267.

(73) Bleday R, Steele Jr G (1991) Second-look surgery for recurrent colorectal carcinoma: is it worthwhile? *Semin Surg Oncol* **7**(3): 171–176.

(74) Blumgart L, Fong Y (1995) Results of medical treatment of patients with colorectal carcinoma metastatic to the liver. *Curr Probl Surg* **32:** 345–360.

(75) Boring CC, Squires TS, Tong T (1991) Cancer statistics, 1991. *CA Cancer J Clin* **41**(1): 19–36.

(76) Boyle CA, Decoufle P (1990) National sources of vital status information: extent of coverage and possible selectivity in reporting. *Am J Epidemiol* **131**(1): 160–168.

(77) Branscheid D, Krysa S, Wollkopf G, Bulzebruck H, Probst G, Horn M *et al* (1992) Does ND-YAG laser extend the indications for resection of pulmonary metastases? *Eur J Cardiothorac Surg* **6**(11): 590–596.

(78) Brown C, Warren S (1938) Visceral metastases from rectal carcinoma. *Surg Gynecol Obstet* **66:** 611–621.

(79) Cahan WG (1973) Excision of melanoma metastases to lung: problems in diagnosis and management. *Ann Surg* **178**(6): 703–709.

(80) Cahan WG, Castro EB, Hajdu SI (1974) Proceedings: the significance of a solitary lung shadow in patients with colon carcinoma. *Cancer* **33**(2): 414–421.

(81) Cahan WG, Castro EB, Hajdu SI (1974) Therapeutic pulmonary resection of colonic carcinoma metastatic to lung. *Dis Colon Rectum* **17**(3): 302–309.

(82) Callery CD, Holmes EC, Vernon S, Huth J, Coulson WF, Skinner DG (1983) Resection of pulmonary metastases from nonseminomatous testicular tumors. Correlation of clinical and histological features with treatment outcome. *Cancer* **51**(6): 1152–1158.

(83) Casciato DA, Nagurka C, Tabbarah HJ (1983) Prolonged survival with unresected pulmonary metastases. *Ann Thorac Surg* **36**(2): 202–208.

(84) Cedermark BJ, Blumenson LE, Pickren JW, Holyoke DE, Elias EG (1977) The significance of metastases to the adrenal glands in adenocarcinoma of the colon and rectum. *Surg Gynecol Obstet* **144**(4): 537–546.

(85) Cerdan F, Diez M, Muguerza J, Torres A, Suarez A, Balibrea J (1992) Tratamiento quirurgico de las metastasis pulmonares del cancer colorectal. *Cir Esp* **51:** 34–38.

(86) Chang AE, Schaner EG, Conkle DM, Flye MW, Doppman JL, Rosenberg SA (1979) Evaluation of computed tomography in the detection of pulmonary metastases: a prospective study. *Cancer* **43**(3): 913–916.

(87) Cheng LC, Sham JS, Chiu CS, Fu KH, Lee JW, Mok CK (1996) Surgical resection of pulmonary metastases from nasopharyngeal carcinoma. *Aust N Z J Surg* **66**(2): 71–73.

(88) Choksi LB, Takita H, Vincent RG (1972) The surgical management of solitary pulmonary metastasis. *Surg Gynecol Obstet* **134**(3): 479–482.

(89) Cliffon E, Dasgupta T, Pool J (1964) Bilateral pulmonary resection for primary or metastatic lung cancer. *Cancer* **17:** 86–94.

(90) Collins V, Loeffler R, Tivey H (1956) Observations on growth rates of human tumors. *Am J Roentgenol Radium Ther Nucl Med* **76**(5): 988–1000.

(91) Collins V (1962) Time of occurrence of pulmonary metastasis from carcinoma of colon and rectum. *Cancer* **15:** 387–395.

(92) Compton CC (1999) Pathology report in colon cancer: what is prognostically important? *Dig Dis* **17**(2): 67–79.

(93) Conti JA, Kemeny NE, Saltz LB, Huang Y, Tong WP, Chou TC *et al* (1996) Irinotecan is an active agent in untreated patients with metastatic colorectal cancer. *J Clin Oncol* **14**(3): 709–715.

(94) Cunningham JD, Fong Y, Shriver C, Melendez J, Marx WL, Blumgart LH (1994) One hundred consecutive hepatic resections. Blood loss, transfusion, and operative technique. *Arch Surg* **129**(10): 1050–1056.

(95) de Brauw LM, van de Velde CJ, Bouwhuis-Hoogerwerf ML, Zwaveling A (1987) diagnostic evaluation and survival analysis of colorectal cancer patients with liver metastases. *J Surg Oncol* **34**(2): 81–86.

(96) de Gramont A, Figer A, Seymour M, Homerin M, Hmissi A, Cassidy J *et al* (2000) Leucovorin and fluorouracil with or without oxaliplatin as first-line treatment in advanced colorectal cancer. *J Clin Oncol* **18**(16): 2938–2947.

(97) Dionne L (1965) The pattern of blood-borne metastasis from carcinoma of the rectum. *Cancer* **18:** 775–781.

(98) Divis G (1927) Ein bertrag zur operativen behandlung der lungen-geschwulste. *Acta Chir Scand* **62:** 329–341.

(99) Doci R, Gennari L, Bignami P, Montalto F, Morabito A, Bozzetti F (1991) One hundred patients with hepatic metastases from colorectal cancer treated by resection: analysis of prognostic determinants. *Br J Surg* **78**(7): 797–801.

(100) Dowling RD, Wachs ME, Ferson PF, Landreneau RJ (1992) Thoracoscopic neodymium: yttrium aluminum garnet laser resection of a pulmonary metastasis. *Cancer* **70**(7): 1873–1875.

(101) Dowling RD, Keenan RJ, Ferson PF, Landreneau RJ (1993) Video-assisted thoracoscopic resection of pulmonary metastases. *Ann Thorac Surg* **56**(3): 772–775.

(102) Dowling RD, Landreneau RJ, Miller DL (1998) Video-assisted thoracoscopic surgery for resection of lung metastases. *Chest* **113**(1 Suppl): 2S–5S.

(103) Downey RJ, McCormack P, LoCicero J, III (1996) dissemination of malignant tumors after video-assisted thoracic surgery: a report of twenty-one cases. The Video-Assisted Thoracic Surgery Study Group. *J Thorac Cardiovasc Surg* **111**(5): 954–960.

(104) Dwight RW, Higgins GA, Keehn RJ (1969) Factors influencing survival after resection in cancer of the colon and rectum. *Am J Surg* **117**(4): 512–522.

(105) Dwight RW, Humphrey EW, Higgins GA, Keehn RJ (1973) FUDR as an adjuvant to surgery in cancer of the large bowel. *J Surg Oncol* **5**(3): 243–249.

(106) Ehrenhaft JL (1951) Pulmonary resections for metastatic lesions. *AMA Arch Surg* **63**(3): 326–336.

(107) Ehrenhaft JL, Lawrence M, Sensenig D (1958) Pulmonary resections for metastatic lesions. *AMA Arch Surg* **77**(4): 606–612.

(108) El-Domeiri AA (1980) Treatment of hepatic metastases in cancer of the colon and rectum. A preliminary report. *Cancer* **45**(9): 2245–2248.

(109) Elias D, Rougier P, Mankarios H, Fahrat F, Lasser P (1993) (Resectable liver metastases and synchronous extra-hepatic sites of colorectal origin. Surgical indications). *Presse Med* **22**(11): 515–520.

(110) Ercan S, Nichols FC, III, Trastek VF, Deschamps C, Allen MS, Miller DL *et al* (2004) Prognostic significance of lymph node metastasis found during pulmonary metastasectomy for extrapulmonary carcinoma. *Ann Thorac Surg* **77**(5): 1786–1791.

(111) Fallon RH, Roper CL (1967) Operative treatment of metastatic pulmonary cancer. *Ann Surg* **166**(2): 263–265.

(112) Farrell J (1935) Pulmonary metastases: a pathological, clinical, roentgenologic study based on 78 cases of necropsy. *Radiology* **24:** 444–451.

(113) Ferson PF, Keenan RJ, Luketich JD (1998) The role of video-assisted thoracic surgery in pulmonary metastases. *Chest Surg Clin N Am* **8**(1): 59–76.

(114) Fidler IJ, Nicolson GL (1977) Fate of recirculating B16 melanoma metastatic variant cells in parabiotic syngeneic recipients. *J Natl Cancer Inst* **58**(6): 1867–1872.

(115) Floyd CE, Stirling CT, Cohn Jr I (1966) Cancer of the colon, rectum and anus: review of 1,687 cases. *Ann Surg* **163**(6): 829–837.

(116) Flye MW, Woltering G, Rosenberg SA (1984) Aggressive pulmonary resection for metastatic osteogenic and soft tissue sarcomas. *Ann Thorac Surg* **37**(2): 123–127.

(117) Fong Y, Blumgart LH, Cohen AM (1995) Surgical treatment of colorectal metastases to the liver. *CA Cancer J Clin* **45**(1): 50–62.

(118) Fong Y, Cohen AM, Fortner JG, Enker WE, Turnbull AD, Coit DG *et al* (1997) Liver resection for colorectal metastases. *J Clin Oncol* **15**(3): 938–946.

(119) Fong Y, Fortner J, Sun RL, Brennan MF, Blumgart LH (1999) Clinical score for predicting recurrence after hepatic resection for metastatic colorectal cancer: analysis of 1001 consecutive cases. *Ann Surg* **230**(3): 309–318.

(120) Fortner JG, Silva JS, Golbey RB, Cox EB, Maclean BJ (1984) Multivariate analysis of a personal series of 247 consecutive patients with liver metastases from colorectal cancer. I. Treatment by hepatic resection. *Ann Surg* **199**(3): 306–316.

(121) Fortner JG (1988) Recurrence of colorectal cancer after hepatic resection. *Am J Surg* **155**(3): 378–382.

(122) Fried G, Tsalik M, Stein M, Dale J, Haim N (1995) 5-Fluorouracil and low-dose leucovorin in metastatic colorectal cancer: a pilot study. *Cancer Chemother Pharmacol* **35**(5): 437–440.

(123) Friedel G, Pastorino U, Ginsberg RJ, Goldstraw P, Johnston M, Pass H *et al* (2002) Results of lung metastasectomy from breast cancer: prognostic criteria on the basis of 467 cases of the International Registry of Lung Metastases. *Eur J Cardiothorac Surg* **22**(3): 335–344.

(124) Fujisawa T, Yamaguchi Y, Saitoh Y, Yusa T, Urabe N, Shiba M *et al* (1991) Surgical treatment of metastatic lung tumours. *Surg Ther* **65:** 367–372.

(125) Fuller Jr AF, Scannell JG, Wilkins Jr EW (1985) Pulmonary resection for metastases from gynecologic cancers: Massachusetts General Hospital experience, 1943–1982. *Gynecol Oncol* **22**(2): 174–180.

(126) Gastrointestinal Tumor Study Group (1984) Adjuvant therapy of colon cancer—results of a prospectively randomized trial. *N Engl J Med* **310**(12): 737–743.

(127) Gatta G, Faivre J, Capocaccia R, Ponz de LM (1998) Survival of colorectal cancer patients in Europe during the period 1978–1989. EUROCARE Working Group. *Eur J Cancer* **34**(14 Spec No): 2176–2183.

(128) Giacchetti S, Itzhaki M, Gruia G, Adam R, Zidani R, Kunstlinger F *et al* (1999) Long-term survival of patients with unresectable colorectal cancer liver metastases following infusional chemotherapy with 5-fluorouracil, leucovorin, oxaliplatin and surgery. *Ann Oncol* **10**(6): 663–669.

(129) Giacchetti S, Perpoint B, Zidani R, Le BN, Faggiuolo R, Focan C *et al* (2000) Phase III multicenter randomized trial of oxaliplatin added to chronomodulated fluorouracil-leucovorin as first-line treatment of metastatic colorectal cancer. *J Clin Oncol* **18**(1): 136–147.

(130) Gilbert JM, Jeffrey I, Evans M, Kark AE (1984) Sites of recurrent tumour after ‘curative’ colorectal surgery: implications for adjuvant therapy. *Br J Surg* **71**(3): 203–205.

(131) Girard P, Baldeyrou P, Le CT, Le CA, Brigandi A, Grunenwald D (1994) Surgery for pulmonary metastases. Who are the 10-year survivors? *Cancer* **74**(10): 2791–2797.

(132) Girard P, Baldeyrou P, Grunenwald D (1995) (Lung metastases from colorectal cancer: results of surgery). *Presse Med* **24**(22): 1028–1032.

(133) Gliedman M, Horowitz S, Lewis F (1957) Lung resection for metastatic cancer; 29 cases from the University of Minnesota and a collected review of 264 cases. *Surgery* **42**(3): 521–532.

(134) Goldberg RM, Fleming TR, Tangen CM, Moertel CG, Macdonald JS, Haller DG *et al* (1998) Surgery for recurrent colon cancer: strategies for identifying resectable recurrence and success rates after resection. Eastern Cooperative Oncology Group, the North Central Cancer Treatment Group, and the Southwest Oncology Group. *Ann Intern Med* **129**(1): 27–35.

(135) Goldberg RM, Sargent DJ, Morton RF, Fuchs CS, Ramanathan RK, Williamson SK *et al* (2004) A randomized controlled trial of fluorouracil plus leucovorin, irinotecan, and oxaliplatin combinations in patients with previously untreated metastatic colorectal cancer. *J Clin Oncol* **22**(1): 23–30.

(136) Goorin AM, Delorey MJ, Lack EE, Gelber RD, Price K, Cassady JR *et al* (1984) Prognostic significance of complete surgical resection of pulmonary metastases in patients with osteogenic sarcoma: analysis of 32 patients. *J Clin Oncol* **2**(5): 425–431.

(137) Gossot D, Miaux Y, Guermazi A, Celerier M, Friga J (1994) The hook-wire technique for localization of pulmonary nodules during thoracoscopic resection. *Chest* **105**(5): 1467–1469.

(138) Gough DB, Donohue JH, Trastek VA, Nagorney DM (1994) Resection of hepatic and pulmonary metastases in patients with colorectal cancer. *Br J Surg* **81**(1): 94–96.

(139) Gray BN (1980) Colorectal cancer: the natural history of disseminated disease – a review. *Aust N Z J Surg* **50**(6): 643–646.

(140) Griffith KD, Sugarbaker PH, Chang AE (1990) Repeat hepatic resections for colorectal metastases. *Surgery* **107**(1): 101–104.

(141) Groeger AM, Kandioler D, Mueller MR, End A, Eckersberger F, Wolner E (1997) Survival after surgical treatment of recurrent pulmonary metastases. *Eur J Cardiothorac Surg* **12**(5): 703–705.

(142) Groves LK, Effler DB (1956) Surgery for metastatic neoplastic disease in the lung; review of 38 cases. *Cleve Clin Q* **23**(1): 16–27.

(143) Grunenwald D, Spaggiari L, Girard P, Baldeyrou P, Posea R, Lamer C *et al* (1997) Lung resection for recurrence after pneumonectomy for metastases. *Bull Cancer* **84**(3): 277–281.

(144) Gutman M, Fidler IJ (1995) Biology of human colon cancer metastasis. *World J Surg* **19**(2): 226–234.

(145) Habein H, Clagett O, McDonald JR (1959) Pulmonary resection for metastatic tumors. *AMA Arch Surg* **78**(5): 716–723.

(146) Hagmar B (1972) Cell surface charge and metastasis formation. A study on the effects of dextrans and heparin on tumour cells and experimental metastases in a syngeneic murine system. *Acta Pathol Microbiol Scand A* **80**(3): 357–366.

(147) Haller DG (1995) An overview of adjuvant therapy for colorectal cancer. *Eur J Cancer* **31A**(7–8): 1255–1263.

(148) Hamy A, Baron O, Bennouna J, Roussel JC, Paineau J, Douillard JY (2001) Resection of hepatic and pulmonary metastases in patients with colorectal cancer. *Am J Clin Oncol* **24**(6): 607–609.

(149) Harmon KE, Ryan Jr JA, Biehl TR, Lee FT (1999) Benefits and safety of hepatic resection for colorectal metastases. *Am J Surg* **177**(5): 402–404.

(150) Harpole Jr DH, Johnson CM, Wolfe WG, George SL, Seigler HF (1992) Analysis of 945 cases of pulmonary metastatic melanoma. *J Thorac Cardiovasc Surg* **103**(4): 743–748.

(151) Hase K, Shatney C, Johnson D, Trollope M, Vierra M (1993) Prognostic value of tumor “budding” in patients with colorectal cancer. *Dis Colon Rectum* **36**(7): 627–635.

(152) Headrick JR, Miller DL, Nagorney DM, Allen MS, Deschamps C, Trastek VF *et al* (2001) Surgical treatment of hepatic and pulmonary metastases from colon cancer. *Ann Thorac Surg* **71**(3): 975–979.

(153) Hermanek Jr P, Wiebelt H, Riedl S, Staimmer D, Hermanek P (1994) (Long-term results of surgical therapy of colon cancer. Results of the Colorectal Cancer Study Group). *Chirurg* **65**(4): 287–297.

(154) Hermanek P (1995) pTNM and residual tumor classifications: problems of assessment and prognostic significance. *World J Surg* **19**(2): 184–190.

(155) Higashiyama M, Kodama K, Takami K, Higaki N, Yokouchi H, Nakayama T *et al* (2002) Intraoperative lavage cytologic analysis of surgical margins as a predictor of local recurrence in pulmonary metastasectomy. *Arch Surg* **137**(4): 469–474.

(156) Higgins G, Lee L, Dwight R (1978) The case for adjuvant fluorouracil in colorectal cancer. *Cancer Clin Trials* **1:** 35–41.

(157) Higgins GA (1983) Current status of adjuvant therapy in the treatment of large bowel cancer. *Surg Clin North Am* **63**(1): 137–150.

(158) Holmes EC, Ramming KP, Eilber FR, Morton DL (1977) The surgical management of pulmonary metastases. *Semin Oncol* **4**(1): 65–69.

(159) Hood R, McBurney R, Clagett O (1955) Metastatic malignant lesions of the lungs treated by pulmonary resection. *J Thorac Surg* **30**(1): 81–89.

(160) Hughes ES, McConchie IH, McDermott FT, Johnson WR, Price AB (1982) Resection of lung metastases in large bowel cancer. *Br J Surg* **69**(7): 410–412.

(161) Hughes KS, Rosenstein RB, Songhorabodi S, Adson MA, Ilstrup DM, Fortner JG *et al* (1988) Resection of the liver for colorectal carcinoma metastases. A multi-institutional study of long-term survivors. *Dis Colon Rectum* **31**(1): 1–4.

(162) Ike H, Sadahiro S, Oya K, Otani Y, Shimada H, Yamaguchi S *et al* (2001) (Combination chemotherapy of doxifluridine plus mitomycin C for colorectal lung metastasis—phase II study). *Gan To Kagaku Ryoho* **28**(9): 1263–1268.

(163) Ike H, Shimada H, Ohki S, Togo S, Yamaguchi S, Ichikawa Y (2002) Results of aggressive resection of lung metastases from colorectal carcinoma detected by intensive follow-up. *Dis Colon Rectum* **45**(4): 468–473.

(164) Impact Investigators (1995) Efficacy of adjuvant fluorouracil and folinic acid in colon cancer. International Multicentre Pooled Analysis of Colon Cancer Trials (IMPACT) investigators. *Lancet* **345**(8955): 939–944.

(165) Inoue M, Kotake Y, Nakagawa K, Fujiwara K, Fukuhara K, Yasumitsu T (2000) Surgery for pulmonary metastases from colorectal carcinoma. *Ann Thorac Surg* **70**(2): 380–383.

(166) Iwatsuki S, Esquivel CO, Gordon RD, Starzl TE (1986) Liver resection for metastatic colorectal cancer. *Surgery* **100**(4): 804–810.

(167) Jablons D, Steinberg SM, Roth J, Pittaluga S, Rosenberg SA, Pass HI (1989) Metastasectomy for soft tissue sarcoma. Further evidence for efficacy and prognostic indicators. *J Thorac Cardiovasc Surg* **97**(5): 695–705.

(168) Jauch KW, Koebe HG, Piltz S, Hertlein H, Dienemann H (1993) (Surgery of metastases—can surgery of metastases be radical?). *Zentralbl Chir* **118**(9): 508–515.

(169) Jensick R, van Hazel W (1958) Appraisal of progress in surgical therapy. *Surgery* **43**(6): 1002–1020.

(170) Jett JR, Hollinger CG, Zinsmeister AR, Pairolero PC (1983) Pulmonary resection of metastatic renal cell carcinoma. *Chest* **84**(4): 442–445.

(171) Johnston MR (1983) Median sternotomy for resection of pulmonary metastases. *J Thorac Cardiovasc Surg* **85**(4): 516–522.

(172) Joseph WL, Morton DL, Adkins PC (1971) Variation in tumor doubling time in patients with pulmonary metastatic disease. *J Surg Oncol* **3**(2): 143–149.

(173) Judson WF, Harbrecht PJ, Fry DE (1983) Associated lung lesions in patients with primary head and neck carcinoma. *Am Surg* **49**(9): 487–491.

(174) Kamiyoshihara M, Hirai T, Kawashima O, Ishikawa S, Morishita Y (1998) The surgical treatment of metastatic tumors in the lung: is lobectomy with mediastinal lymph node dissection suitable treatment? *Oncol Rep* **5**(2): 453–457.

(175) Kandioler D, Kromer E, Tuchler H, End A, Muller MR, Wolner E *et al* (1998) Long-term results after repeated surgical removal of pulmonary metastases. *Ann Thorac Surg* **65**(4): 909–912.

(176) Karapetis CS, Yip D, Harper PG (1999) The treatment of metastatic colorectal cancer. *Int J Clin Pract* **53**(4): 287–294.

(177) Kelly C, Langston H (1956) The treatment of metastatic pulmonary malignancy. *J Thorac Surg* **31**(3): 298–315.

(178) Kligerman MM (1975) Preoperative radiation theration therapy in rectal cancer. *Cancer* **36**(2): 691–695.

(179) Kobayashi K, Kawamura M, Ishihara T (1999) Surgical treatment for both pulmonary and hepatic metastases from colorectal cancer. *J Thorac Cardiovasc Surg* **118**(6): 1090–1096.

(180) Koehne CH, Midgley R, Seymour M, Kerr DJ (1999) Advanced colorectal cancer: which regimes should we recommend? *Ann Oncol* **10**(8): 877–882.

(181) Kohne CH, Cunningham D, Di CF, Glimelius B, Blijham G, Aranda E *et al* (2002) Clinical determinants of survival in patients with 5-fluorouracil-based treatment for metastatic colorectal cancer: results of a multivariate analysis of 3825 patients. *Ann Oncol* **13**(2): 308–317.

(182) Kokudo N, Miki Y, Sugai S, Yanagisawa A, Kato Y, Sakamoto Y *et al* (2002) Genetic and histological assessment of surgical margins in resected liver metastases from colorectal carcinoma: minimum surgical margins for successful resection. *Arch Surg* **137**(7): 833–840.

(183) Kondo D, Goya T, Kondo H, Tsuchiya R, Naruke T, Suemasu K (1989) (Surgical treatment in pulmonary metastases of colorectal cancer). *Nippon Geka Gakkai Zasshi* **90**(1): 75–81.

(184) Kouri M, Pyrhonen S, Kuusela P (1992) Elevated CA19–9 as the most significant prognostic factor in advanced colorectal carcinoma. *J Surg Oncol* **49**(2): 78–85.

(185) Kune GA, Kune S, Field B, White R, Brough W, Schellenberger R *et al* (1990) Survival in patients with large-bowel cancer. A population-based investigation from the Melbourne Colorectal Cancer Study. *Dis Colon Rectum* **33**(11): 938–946.

(186) Kunishima K, Karasawa K, Takagi I, Suyama M (1982) (Surgical treatment of pulmonary metastasis of colorectal cancer). *Nippon Kyobu Geka Gakkai Zasshi* **30**(8): 1423–1427.

(187) Labow DM, Buell JE, Yoshida A, Rosen S, Posner MC (2002) Isolated pulmonary recurrence after resection of colorectal hepatic metastases—is resection indicated? *Cancer J* **8**(4): 342–347.

(188) Landreneau RJ, De Giacomo T, Mack MJ, Hazelrigg SR, Ferson PF, Keenan RJ *et al* (2000) Therapeutic video-assisted thoracoscopic surgical resection of colorectal pulmonary metastases. *Eur J Cardiothorac Surg* **18**(6): 671–676.

(189) Langer B (1985) Colorectal cancer: managing distant metastases. *Can J Surg* **28**(5): 419–421.

(190) Launoy G, Grosclaude P, Pienkowski P, Faivre J, Menegoz F, Schaffer P *et al* (1992) (Digestive cancers in France. Comparison of the incidence in 7 departments and estimation of incidence in the entire country of France). *Gastroenterol Clin Biol* **16**(8–9): 633–638.

(191) Lehnert T, Knaebel HP, Duck M, Bulzebruck H, Herfarth C (1999) Sequential hepatic and pulmonary resections for metastatic colorectal cancer. *Br J Surg* **86**(2): 241–243.

(192) Lin JC, Wiechmann RJ, Szwerc MF, Hazelrigg SR, Ferson PF, Naunheim KS *et al* (1999) diagnostic and therapeutic video-assisted thoracic surgery resection of pulmonary metastases. *Surgery* **126**(4): 636–641.

(193) Liu HP, Lin PJ, Hsieh MJ, Chang JP, Chang CH (1995) Application of thoracoscopy for lung metastases. *Chest* **107**(1): 266–268.

(194) Loehe F, Kobinger S, Hatz RA, Helmberger T, Loehrs U, Fuerst H (2001) Value of systematic mediastinal lymph node dissection during pulmonary metastasectomy. *Ann Thorac Surg* **72**(1): 225–229.

(195) Logan SE, Meier SJ, Ramming KP, Morton DL, Longmire Jr WP (1982) Hepatic resection of metastatic colorectal carcinoma: a ten-year experience. *Arch Surg* **117**(1): 25–28.

(196) Loprinzi CL (1995) Follow-up testing for curatively treated cancer survivors. What to do? *JAMA* **273**(23): 1877–1878.

(197) Mack MJ, Shennib H, Landreneau RJ, Hazelrigg SR (1993) Techniques for localization of pulmonary nodules for thoracoscopic resection. *J Thorac Cardiovasc Surg* **106**(3): 550–553.

(198) Maebeya S, Miyoshi S, Suzuma T, Hirai I, Yoshimasu T, Naito Y (1993) [Surgical resection of pulmonary metastases from colorectal cancer]. *Kyobu Geka* **46**(6): 503–506.

(199) Malcolm AW, Perencevich NP, Olson RM, Hanley JA, Chaffey JT, Wilson RE (1981) Analysis of recurrence patterns following curative resection for carcinoma of the colon and rectum. *Surg Gynecol Obstet* **152**(2): 131–136.

(200) Mandelbaum I, Yaw PB, Einhorn LH, Williams SD, Rowland RG, Donohue JP (1983) The importance of one-stage median sternotomy and retroperitoneal node dissection in disseminated testicular cancer. *Ann Thorac Surg* **36**(5): 524–528.

(201) Marcove R, Huvos A (1971) Osteogenic sarcoma in childhood. *N Y State J Med* **71:** 857–859.

(202) Marion J, Burgers V, Breur K, van Dobbenburgh OA, Hazebroek F, Vos A *et al* (1980) Role of metastatectomy without chemotherapy in the management of osteosarcoma in children. *Cancer* **45**(7): 1664–1668.

(203) Marks P, Ferrag MZ, Ashraf H (1981) Rationale for the surgical treatment of pulmonary metastases. *Thorax* **36**(9): 679–682.

(204) Marshall JL (2001) Capecitabine in colorectal cancer. *Oncology (Williston Park)* **15**(1 Suppl 2): 41–46.

(205) Martini N, Huvos AG, Mike V, Marcove RC, Beattie Jr EJ (1971) Multiple pulmonary resections in the treatment of osteogenic sarcoma. *Ann Thorac Surg* **12**(3): 271–280.

(206) Martini N, McCormack PM (1998) Evolution of the surgical management of pulmonary metastases. *Chest Surg Clin N Am* **8**(1): 13–27.

(207) Masters GA, Golomb HM (1995) Management of pulmonary metastases. *Lancet* **346**(8967): 68.

(208) Matthay RA, Arroliga AC (1993) Resection of pulmonary metastases. *Am Rev Respir Dis* **148**(6 Pt 1): 1691–1696.

(209) Mayo C, Schlicke C (1978) Carcinoma of the colon and rectum: a decade of experience at the Lahey Clinic. *Dis Colon Rectum* **22:** 717–720.

(210) McCormack PM, Bains MS, Beattie Jr EJ, Martini N (1978) Pulmonary resection in metastatic carcinoma. *Chest* **73**(2): 163–166.

(211) McCormack PM, Attiyeh FF (1979) Resected pulmonary metastases from colorectal cancer. *Dis Colon Rectum* **22**(8): 553–556.

(212) McCormack PM, Martini N (1979) The changing role of surgery for pulmonary metastases. *Ann Thorac Surg* **28**(2): 139–145.

(213) McCormack PM, Ginsberg KB, Bains MS, Burt ME, Martini N, Rusch VW *et al* (1993) Accuracy of lung imaging in metastases with implications for the role of thoracoscopy. *Ann Thorac Surg* **56**(4): 863–865.

(214) McCormack PM, Bains MS, Begg CB, Burt ME, Downey RJ, Panicek DM *et al* (1996) Role of video-assisted thoracic surgery in the treatment of pulmonary metastases: results of a prospective trial. *Ann Thorac Surg* **62**(1): 213–216.

(215) McCormack PM, Ginsberg RJ (1998) Current management of colorectal metastases to lung. *Chest Surg Clin N Am* **8**(1): 119–126.

(216) Meyer WH, Schell MJ, Kumar AP, Rao BN, Green AA, Champion J *et al* (1987) Thoracotomy for pulmonary metastatic osteosarcoma. An analysis of prognostic indicators of survival. *Cancer* **59**(2): 374–379.

(217) Midgley R, Kerr D (1999) Colorectal cancer. *Lancet* **353**(9150): 391–399.

(218) Millikan KW, Staren ED, Doolas A (1997) Invasive therapy of metastatic colorectal cancer to the liver. *Surg Clin North Am* **77**(1): 27–48.

(219) Minagawa M, Makuuchi M, Torzilli G, Takayama T, Kawasaki S, Kosuge T *et al* (2000) Extension of the frontiers of surgical indications in the treatment of liver metastases from colorectal cancer: long-term results. *Ann Surg* **231**(4): 487–499.

(220) Mineo TC, Ambrogi V, Paci M, Iavicoli N, Pompeo E, Nofroni I (2001) Transxiphoid bilateral palpation in video-assisted thoracoscopic lung metastasectomy. *Arch Surg* **136**(7): 783–788.

(221) Minnard E, Fong Y, Weigel T, Fortner J, Blumgart L, Burt M (1996) Surgical resection for hepatic and pulmonary colorectal metastases. *Proc Am Soc Clin Oncol* **15:** 552–557.

(222) Moersch R, Clagett O (1961) Pulmonary resection for metastatic tumors of the lungs. *Surgery* **50:** 579–585.

(223) Moertel CG, Fleming TR, Macdonald JS, Haller DG, Laurie JA, Goodman PJ *et al* (1990) Levamisole and fluorouracil for adjuvant therapy of resected colon carcinoma. *N Engl J Med* **322**(6): 352–358.

(224) Moertel CG, Fleming TR, Macdonald JS, Haller DG, Laurie JA, Tangen CM *et al* (1995) Fluorouracil plus levamisole as effective adjuvant therapy after resection of stage III colon carcinoma: a final report. *Ann Intern Med* **122**(5): 321–326.

(225) Monteiro A, Arce N, Bernardo J, Eugenio L, Antunes MJ (2004) Surgical resection of lung metastases from epithelial tumors. *Ann Thorac Surg* **77**(2): 431–437.

(226) Morrow CE, Vassilopoulos PP, Grage TB (1980) Surgical resection for metastatic neoplasms of the lung: experience at the University of Minnesota Hospitals. *Cancer* **45**(12): 2981–2985.

(227) Morrow CE, Grage TB, Sutherland DE, Najarian JS (1982) Hepatic resection for secondary neoplasms. *Surgery* **92**(4): 610–614.

(228) Mountain CF, Khalil KG, Hermes KE, Frazier OH (1978) The contribution of surgery to the management of carcinomatous pulmonary metastases. *Cancer* **41**(3): 833–840.

(229) Mountain CF, McMurtrey MJ, Hermes KE (1984) Surgery for pulmonary metastasis: a 20-year experience. *Ann Thorac Surg* **38**(4): 323–330.

(230) Muhe E, Gall FP, Angermann B (1981) Surgical treatment of metastases to the lung and liver. *Surg Gynecol Obstet* **152**(2): 211–214.

(231) Murata S, Moriya Y, Akasu T, Fujita S, Sugihara K (1998) Resection of both hepatic and pulmonary metastases in patients with colorectal carcinoma. *Cancer* **83**(6): 1086–1093.

(232) Murphy P, Alexander P, Kirkham N, Fleming J, Taylor I (1986) Pattern of spread of blood-borne tumour. *Br J Surg* **73**(10): 829–834.

(233) Murthy SC, Kim K, Rice TW, Rajeswaran J, Bukowski R, DeCamp MM *et al* (2005) Can we predict long-term survival after pulmonary metastasectomy for renal cell carcinoma? *Ann Thorac Surg* **79**(3): 996–1003.

(234) Muto T, Kotake K, Koyama Y (2001) Colorectal cancer statistics in Japan: data from JSCCR registration, 1974–1993. *Int J Clin Oncol* **6**(4): 171–176.

(235) Mutsaerts EL, Zoetmulder FA, Meijer S, Baas P, Hart AA, Rutgers EJ (2001) Outcome of thoracoscopic pulmonary metastasectomy evaluated by confirmatory thoracotomy. *Ann Thorac Surg* **72**(1): 230–233.

(236) Mutsaerts EL, Zoetmulder FA, Meijer S, Baas P, Hart AA, Rutgers EJ (2002) Long-term survival of thoracoscopic metastasectomy vs metastasectomy by thoracotomy in patients with a solitary pulmonary lesion. *Eur J Surg Oncol* **28**(8): 864–868.

(237) Nagakura S, Shirai Y, Yamato Y, Yokoyama N, Suda T, Hatakeyama K (2001) Simultaneous detection of colorectal carcinoma liver and lung metastases does not warrant resection. *J Am Coll Surg* **193**(2): 153–160.

(238) Nakagawa K, Matsubara T, Seki M, Tsuchiya S, Kinoshita G, Nishi M *et al* (1987) Present status of surgical treatment for metastatic lung tumours. *Jpn J Chest Dis* **46:** 716–724.

(239) Nakagawa K, Tsuchiya S, Seki M, Matsubara T, Ohta H (1989) Surgical treatment of metastatic tumor from colorectal cancer. *Shujyutsu* **43:** 1699–1705.

(240) Nathan M, Collins V, Dams R (1962) differentiation of benign and malignant pulmonary nodules by growth rate. *Radiology* **79:** 221–232.

(241) Nelson W, Brown R, Baers S (1957) The repeated surgical attack on solitary metastatic neoplasms. *Ann Surg* **146**(5): 790–798.

(242) Newland RC, Chapuis PH, Pheils MT, MacPherson JG (1981) The relationship of survival to staging and grading of colorectal carcinoma: a prospective study of 503 cases. *Cancer* **47**(6): 1424–1429.

(243) Nicolson GL (1982) Cancer metastasis. Organ colonization and the cell-surface properties of malignant cells. *Biochim Biophys Acta* **695**(2): 113–176.

(244) Nomori H, Horio H (1996) Colored collagen is a long-lasting point marker for small pulmonary nodules in thoracoscopic operations. *Ann Thorac Surg* **61**(4): 1070–1073.

(245) Nordlinger B, Quilichini MA, Parc R, Hannoun L, Delva E, Huguet C (1987) Hepatic resection for colorectal liver metastases. Influence on survival of preoperative factors and surgery for recurrences in 80 patients. *Ann Surg* **205**(3): 256–263.

(246) Nordlinger B, Guiguet M, Vaillant JC, Balladur P, Boudjema K, Bachellier P *et al* (1996) Surgical resection of colorectal carcinoma metastases to the liver. A prognostic scoring system to improve case selection, based on 1568 patients. Association Francaise de Chirurgie. *Cancer* **77**(7): 1254–1262.

(247) Ochsner A, Clemmons E, Mitchell W (1963) Treatment of metastatic pulmonary malignant lesions. *J Lancet* **83:** 16–24.

(248) Ohata M (1993) (Surgical treatment for metastatic lung tumors—with special references to colorectal lung metastases). *Hum Cell* **6**(2): 88–93.

(249) Pass HI (1998) Surgical management of pulmonary metastases. *Curr Opin Oncol* **10**(2): 146–150.

(250) Pastorino U, Valente M, Gasparini M, Azzarelli A, Santoro A, Alloisio M *et al* (1989) Lung resection for metastatic sarcomas: total survival from primary treatment. *J Surg Oncol* **40**(4): 275–280.

(251) Pastorino U, Buyse M, Friedel G, Ginsberg RJ, Girard P, Goldstraw P *et al* (1997) Long-term results of lung metastasectomy: prognostic analyses based on 5206 cases. *J Thorac Cardiovasc Surg* **113:** 37–49.

(252) Pastorino U, McCormack PM, Ginsberg RJ (1998) A new staging proposal for pulmonary metastases. The results of analysis of 5206 cases of resected pulmonary metastases. *Chest Surg Clin N Am* **8**(1): 197–202.

(253) Patterson GA, Todd TR, Ilves R, Pearson FG, Cooper JD (1982) Surgical management of pulmonary metastases. *Can J Surg* **25**(1): 102–105.

(254) Payne WS, Clagett O, Harrison E (1962) Surgical management of bilateral malignant lesions of the lung. *J Thorac Cardiovasc Surg* **43:** 279–290.

(255) Petrowsky H, Gonen M, Jarnagin W, Lorenz M, DeMatteo R, Heinrich S *et al* (2002) Second liver resections are safe and effective treatment for recurrent hepatic metastases from colorectal cancer: a bi-institutional analysis. *Ann Surg* **235**(6): 863–871.

(256) Pihl E, Hughes ES, McDermott FT, Johnson WR, Katrivessis H (1987) Lung recurrence after curative surgery for colorectal cancer. *Dis Colon Rectum* **30**(6): 417–419.

(257) Planchard D, Soria JC, Michiels S, Grunenwald D, Validire P, Caliandro R *et al* (2004) Uncertain benefit from surgery in patients with lung metastases from breast carcinoma. *Cancer* **100**(1): 28–35.

(258) Pogrebniak HW, Pass HI (1993) Initial and reoperative pulmonary metastasectomy: indications, technique, and results. *Semin Surg Oncol* **9**(2): 142–149.

(259) Polk Jr HC, Spratt JS (1983) Recurrent cancer of the colon. *Surg Clin North Am* **63**(1): 151–160.

(260) Poon MA, O'Connell MJ, Moertel CG, Wieand HS, Cullinan SA, Everson LK *et al* (1989) Biochemical modulation of fluorouracil: evidence of significant improvement of survival and quality of life in patients with advanced colorectal carcinoma. *J Clin Oncol* **7**(10): 1407–1418.

(261) Potter DA, Glenn J, Kinsella T, Glatstein E, Lack EE, Restrepo C *et al* (1985) Patterns of recurrence in patients with high-grade soft-tissue sarcomas. *J Clin Oncol* **3**(3): 353–366.

(262) Putnam Jr JB, Roth JA, Wesley MN, Johnston MR, Rosenberg SA (1983) Survival following aggressive resection of pulmonary metastases from osteogenic sarcoma: analysis of prognostic factors. *Ann Thorac Surg* **36**(5): 516–523.

(263) Putnam Jr JB, Roth JA, Wesley MN, Johnston MR, Rosenberg SA (1984) Analysis of prognostic factors in patients undergoing resection of pulmonary metastases from soft tissue sarcomas. *J Thorac Cardiovasc Surg* **87**(2): 260–268.

(264) Putnam Jr JB, Roth JA (1990) Prognostic indicators in patients with pulmonary metastases. *Semin Surg Oncol* **6**(5): 291–296.

(265) Putnam Jr JB, Suell DM, Natarajan G, Roth JA (1993) Extended resection of pulmonary metastases: is the risk justified? *Ann Thorac Surg* **55**(6): 1440–1446.

(266) Ratto C, Sofo L, Ippoliti M, Merico M, Doglietto GB, Crucitti F (1998) Prognostic factors in colorectal cancer. Literature review for clinical application. *Dis Colon Rectum* **41**(8): 1033–1049.

(267) Ravikumar TS, Steele Jr G, Kane R, King V (1991) Experimental and clinical observations on hepatic cryosurgery for colorectal metastases. *Cancer Res* **51**(23 Pt 1): 6323–6327.

(268) Regal AM, Reese P, Antkowiak J, Hart T, Takita H (1985) Median sternotomy for metastatic lung lesions in 131 patients. *Cancer* **55**(6): 1334–1339.

(269) Regnard JF, Levasseur P (1995) (Place of surgery in the treatment of lung metastases from colorectal cancers). *Ann Chir* **49**(2): 121–127.

(270) Regnard JF, Nicolosi M, Coggia M, Spaggiari L, Fourquier P, Levi JF *et al* (1995) [Results of surgical treatment of lung metastases from colorectal cancers]. *Gastroenterol Clin Biol* **19**(4): 378–384.

(271) Regnard JF, Grunenwald D, Spaggiari L, Girard P, Elias D, Ducreux M *et al* (1998) Surgical treatment of hepatic and pulmonary metastases from colorectal cancers. *Ann Thorac Surg* **66**(1): 214–218.

(272) Remy-Jardin M, Remy J, Giraud F, Marquette CH (1993) Pulmonary nodules: detection with thick-section spiral CT versus conventional CT. *Radiology* **187**(2): 513–520.

(273) Rizzoni WE, Pass HI, Wesley MN, Rosenberg SA, Roth JA (1986) Resection of recurrent pulmonary metastases in patients with soft-tissue sarcomas. *Arch Surg* **121**(11): 1248–1252.

(274) Robert JH, Ambrogi V, Mermillod B, Dahabreh D, Goldstraw P (1997) Factors influencing long-term survival after lung metastasectomy. *Ann Thorac Surg* **63**(3): 777–784.

(275) Roberts DG, Lepore V, Cardillo G, Dernevik L, Berggren H, Belboul A *et al* (1989) Long-term follow-up of operative treatment for pulmonary metastases. *Eur J Cardiothorac Surg* **3**(4): 292–296.

(276) Robinson BJ, Rice TW, Strong SA, Rybicki LA, Blackstone EH (1999) Is resection of pulmonary and hepatic metastases warranted in patients with colorectal cancer? *J Thorac Cardiovasc Surg* **117**(1): 66–75.

(277) Rosen CB, Nagorney DM, Taswell HF, Helgeson SL, Ilstrup DM, Van Heerden JA *et al* (1992) Perioperative blood transfusion and determinants of survival after liver resection for metastatic colorectal carcinoma. *Ann Surg* **216**(4): 493–504.

(278) Rosen M, Chan L, Beart Jr RW, Vukasin P, Anthone G (1998) Follow-up of colorectal cancer: a meta-analysis. *Dis Colon Rectum* **41**(9): 1116–1126.

(279) Roth JA, Pass HI, Wesley MN, White D, Putnam JB, Seipp C (1986) Comparison of median sternotomy and thoracotomy for resection of pulmonary metastases in patients with adult soft-tissue sarcomas. *Ann Thorac Surg* **42**(2): 134–138.

(280) Roth JA, Beech DJ, Putnam Jr JB, Pollock RE, Patel SR, Fidler IJ *et al* (1996) Treatment of the patient with lung metastases. *Curr Probl Surg* **33**(11): 881–952.

(281) Rusch V, Saltz L, Venkatraman E, Ginsberg R, McCormack P, Burt M *et al* (1994) A phase II trial of pleurectomy/decortication followed by intrapleural and systemic chemotherapy for malignant pleural mesothelioma. *J Clin Oncol* **12**(6): 1156–1163.

(282) Rusch VW (1995) Pulmonary metastasectomy. Current indications. *Chest* **107**(6 Suppl): 322S–331S.

(283) Saltz LB, Cox JV, Blanke C, Rosen LS, Fehrenbacher L, Moore MJ *et al* (2000) Irinotecan plus fluorouracil and leucovorin for metastatic colorectal cancer. Irinotecan Study Group. *N Engl J Med* **343**(13): 905–914.

(284) Sartorelli KH, Partrick D, Meagher Jr DP (1996) Port-site recurrence after thoracoscopic resection of pulmonary metastasis owing to osteogenic sarcoma. *J Pediatr Surg* **31**(10): 1443–1444.

(285) Sauter ER, Bolton JS, Willis GW, Farr GH, Sardi A (1990) Improved survival after pulmonary resection of metastatic colorectal carcinoma. *J Surg Oncol* **43**(3): 135–138.

(286) Scheele J, Stangl R, Altendorf-Hofmann A (1990) Hepatic metastases from colorectal carcinoma: impact of surgical resection on the natural history. *Br J Surg* **77**(11): 1241–1246.

(287) Scheele J, Tendorf-Hofmann A, Stangl R, Groite H, Gall FP (1989) (Resection of lung metastases of colorectal cancer. Indications and indication limits). *Zentralbl Chir* **114**(10): 639–654.

(288) Scheele J, Stangl R, Tendorf-Hofmann A, Gall FP (1991) Indicators of prognosis after hepatic resection for colorectal secondaries. *Surgery* **110**(1): 13–29.

(289) Scheele J, Tendorf-Hofmann A, Grube T, Hohenberger W, Stangl R, Schmidt K (2001) (Resection of colorectal liver metastases. What prognostic factors determine patient selection?). *Chirurg* **72**(5): 547–560.

(290) Schulten MF, Heiskell CA, Shields TW (1976) The incidence of solitary pulmonary metastasis from carcinoma of the large intestine. *Surg Gynecol Obstet* **143**(5): 727–729.

(291) Schwarz RE, Posner MC, Ferson PF, Keenan RJ, Landreneau RJ (1998) Thoracoscopic techniques for the management of intrathoracic metastases. *Results Surg Endosc* **12**(6): 842–845.

(292) Sellors TH (1970) Treatment of isolated pulmonary metastases. *Br Med J* **2**(5704): 253–256.

(293) Shennib H, Bret P (1993) Intraoperative transthoracic ultrasonographic localization of occult lung lesions. *Ann Thorac Surg* **55**(3): 767–769.

(294) Shimada Y, Yoshino M, Wakui A, Nakao I, Futatsuki K, Sakata Y *et al* (1993) Phase II study of CPT-11, a new camptothecin derivative, in metastatic colorectal cancer. CPT-11 Gastrointestinal Cancer Study Group. *J Clin Oncol* **11**(5): 909–913.

(295) Shimada H, Nanko M, Fujii S, Masui H, Togo S, Ike H (1995) Treatment strategies for hepatic metastasis from colorectal cancer. *J Hepatobiliary Pancreatic Surg* **2:** 116–121.

(296) Shirakusa T, Tsutsui M, Motonaga R, Ando K, Kusano T (1988) Resection of metastatic lung tumor: the evaluation of histologic appearance in the lung. *Am Surg* **54**(11): 655–658.

(297) Silverberg E (1985) Cancer statistics, 1985. *CA Cancer J Clin* **35**(1): 19–35.

(298) Silverberg E, Boring CC, Squires TS (1990) Cancer statistics, 1990. *CA Cancer J Clin* **40**(1): 9–26.

(299) Sischy B (1982) The place of radiotherapy in the management of rectal adenocarcinoma. *Cancer* **50**(11 Suppl): 2631–2637.

(300) Smith JW, Fortner JG, Burt M (1992) Resection of hepatic and pulmonary metastases from colorectal cancer. *Surg Oncol* **1**(6): 399–404.

(301) Spaggiari L, Grunenwald DH, Girard P, Solli P, Le CT (1998) Pneumonectomy for lung metastases: indications, risks, and outcome. *Ann Thorac Surg* **66**(6): 1930–1933.

(302) Steele Jr G, Bleday R, Mayer RJ, Lindblad A, Petrelli N, Weaver D (1991) A prospective evaluation of hepatic resection for colorectal carcinoma metastases to the liver: Gastrointestinal Tumor Study Group Protocol 6584. *J Clin Oncol* **9**(7): 1105–1112.

(303) Stone MD, Cady B, Jenkins RL, McDermott WV, Steele Jr GD (1990) Surgical therapy for recurrent liver metastases from colorectal cancer. *Arch Surg* **125**(6): 718–721.

(304) Sugihara K, Hojo K, Moriya Y, Yamasaki S, Kosuge T, Takayama T (1993) Pattern of recurrence after hepatic resection for colorectal metastases. *Br J Surg* **80**(8): 1032–1035.

(305) Takahashi T, Kato T, Kodaira S, Koyama Y, Sakabe T, Tominaga T *et al* (1996) Prognostic factors of colorectal cancer. Results of multivariate analysis of curative resection cases with or without adjuvant chemotherapy. *Am J Clin Oncol* **19**(4): 408–415.

(306) Takita H, Merrin C, Didolkar MS, Douglass HO, Edgerton F (1977) The surgical management of multiple lung metastases. *Ann Thorac Surg* **24**(4): 359–364.

(307) Takita H, Edgerton F, Karakousis C, Douglass Jr HO, Vincent RG, Beckley S (1981) Surgical management of metastases to the lung. *Surg Gynecol Obstet* **152**(2): 191–194.

(308) Tanaka K, Shimada H, Togo S, Ota M, Yamagichi S, Ike H (2001) Is hepatic resection for multiple liver metastases from colorectal carcinoma acceptable treatment? *Hepatogastroenterology* **48**(39): 803–807.

(309) Tatsuta M, Kodama K, Doi O, Iwanaga T, Kurokawa E (1989) (Surgical treatment and indication for metastatic lung tumor). *Nippon Geka Gakkai Zasshi* **90**(4): 598–604.

(310) Temple WJ, Ketcham AS (1980) Surgical management of isolated systemic metastases. *Semin Oncol* **7**(4): 468–480.

(311) Thayer Jr JO, Overholt RH (1985) Metastatic melanoma to the lung: long-term results of surgical excision. *Am J Surg* **149**(4): 558–562.

(312) Thomford NR, Woolner L, Clagett O (1965) The surgical treatment of metastatic tumours in the lung. *J Thorac Cardiovasc Surg* **49:** 357–363.

(313) Todd T (1997) The surgical treatment of pulmonary metastases. *Chest* **112:** 287S–290S.

(314) Togo S, Fujii S, Yamaguchi S, Ike H, Ooki S, Shimada H (1996) Thoracoscopic lung resection for lung metastasis of colorectal cancer. *Surg Laparosc Endosc* **6**(6): 480–484.

(315) Tournigand C, Andre T, Achille E, Lledo G, Flesh M, Mery-Mignard D *et al* (2004) FOLFIRI followed by FOLFOX6 or the reverse sequence in advanced colorectal cancer: a randomized GERCOR study. *J Clin Oncol* **22**(2): 229–237.

(316) Turk PS, Wanebo HJ (1993) Results of surgical treatment of nonhepatic recurrence of colorectal carcinoma. *Cancer* **71**(12 Suppl): 4267–4277.

(317) Ueno H, Mochizuki H (1997) Clinical significance of extrabowel skipped cancer infiltration in rectal cancer. *Surg Today* **27**(7): 617–622.

(318) Ueno H, Mochizuki H, Tamakuma S (1998) Prognostic significance of extranodal microscopic foci discontinuous with primary lesion in rectal cancer. *Dis Colon Rectum* **41**(1): 55–61.

(319) Ueno H, Mochizuki H, Hatsuse K, Hase K, Yamamoto T (2000) Indicators for treatment strategies of colorectal liver metastases. *Ann Surg* **231**(1): 59–66.

(320) van Dongen JA, van Slooten EA (1978) The surgical treatment of pulmonary metastases. *Cancer Treat Rev* **5**(1): 29–48.

(321) van Geel AN, Pastorino U, Jauch KW, Judson IR, van CF, Buesa JM *et al* (1996) Surgical treatment of lung metastases: The European Organization for Research and Treatment of Cancer-Soft Tissue and Bone Sarcoma Group Study of 255 patients. *Cancer* **77**(4): 675–682.

(322) Vigneswaran WT (1996) Management of pulmonary metastases from colorectal cancer. *Semin Surg Oncol* **12**(4): 264–266.

(323) Virgo KS, Wade TP, Longo WE, Coplin MA, Vernava AM, Johnson FE (1995) Surveillance after curative colon cancer resection: practice patterns of surgical subspecialists. *Ann Surg Oncol* **2**(6): 472–482.

(324) Wade TP, Radford DM, Virgo KS, Johnson FE (1994) Complications and outcomes in the treatment of pancreatic adenocarcinoma in the United States veteran. *J Am Coll Surg* **179**(1): 38–48.

(325) Wagner JS, Adson MA, Van Heerden JA, Adson MH, Ilstrup DM (1984) The natural history of hepatic metastases from colorectal cancer. A comparison with resective treatment. *Ann Surg* **199**(5): 502–508.

(326) Walsh GL, Nesbitt JC (1995) Tumor implants after thoracoscopic resection of a metastatic sarcoma. *Ann Thorac Surg* **59**(1): 215–216.

(327) Wanebo HJ, Koness RJ, Vezeridis MP, Cohen SI, Wrobleski DE (1994) Pelvic resection of recurrent rectal cancer. *Ann Surg* **220**(4): 586–595.

(328) Weiss L (1980) Cancer cell traffic from the lungs to the liver: an example of metastatic inefficiency. *Int J Cancer* **25**(3): 385–392.

(329) Weiss L, Ward PM, Holmes JC (1983) Liver-to-lung traffic of cancer cells. *Int J Cancer* **32**(1): 79–83.

(330) Weiss L, Mayhew E (1985) An approach to the therapy of metastases from cancer of the upper rectum: a working hypothesis. *Cancer Drug Deliv* **2**(1): 19–33.

(331) Weiss L, Grundmann E, Torhorst J, Hartveit F, Moberg I, Eder M *et al* (1986) Haematogenous metastatic patterns in colonic carcinoma: an analysis of 1541 necropsies. *J Pathol* **150**(3): 195–203.

(332) Welch JP, Donaldson GA (1978) detection and treatment of recurrent cancer of the colon and rectum. *Am J Surg* **135**(4): 505–511.

(333) Welch JP, Donaldson GA (1979) The clinical correlation of an autopsy study of recurrent colorectal cancer. *Ann Surg* **189**(4): 496–502.

(334) Wilkins Jr EW, Burke JF, Head JM (1961) The surgical management of metastatic neoplasms in the lung. *J Thorac Cardiovasc Surg* **42:** 298–309.

(335) Wilkins Jr EW, Head JM, Burke JF (1978) Pulmonary resection for metastatic neoplasms in the lung. Experience at the Massachusetts General Hospital. *Am J Surg* **135**(4): 480–483.

(336) Wilson SM, Adson MA (1976) Surgical treatment of hepatic metastases from colorectal cancers. *Arch Surg* **111**(4): 330–334.

(337) Windschitl H, Scott M, Schutt A, McCormack G, Everson L, Cullinan S *et al* (1983) Randomized phase II studies in advanced colorectal carcinoma: a North Central Cancer Treatment Group Study. *Cancer Treat Rep* **67**(11): 1001–1008.

(338) Wong JH, Euhus DM, Morton DL (1988) Surgical resection for metastatic melanoma to the lung. *Arch Surg* **123**(9): 1091–1095.

(339) Woodington G, Waugh J (1963) Results of resection of metastatic tumors of the liver. *Am J Surg* **105:** 24–29.

(340) Wright JO, III, Brandt B, III, Ehrenhaft JL (1982) Results of pulmonary resection for metastatic lesions. *J Thorac Cardiovasc Surg* **83**(1): 94–99.

(341) Yamada H, Katoh H, Kondo S, Okushiba S, Morikawa T (2002) Surgical treatment of pulmonary recurrence after hepatectomy for colorectal liver metastases. *Hepatogastroenterology* **49**(46): 976–979.

(342) Yamamoto J, Kosuge T, Shimada K, Yamasaki S, Moriya Y, Sugihara K (1999) Repeat liver resection for recurrent colorectal liver metastases. *Am J Surg* **178**(4): 275–281.

(343) Yamasaki S, Hasegawa H, Makuuchi M, Koyama Y, Hojo K, Moriya N (1985) Hepatectomy for metastatic liver tumor. *Jpn J Clin Oncol* **15**(1): 121–131.

(344) Yano T, Fukuyama Y, Yokoyama H, Tanaka Y, Miyagi J, Kuninaka S *et al* (1997) Failure in resection of multiple pulmonary metastases from colorectal cancer. *J Am Coll Surg* **185**(2): 120–122.
